# Potential Savings in DHW Facilities through the Use of Solar Thermal Energy in the Hospitals of Extremadura (Spain)

**DOI:** 10.3390/ijerph17082658

**Published:** 2020-04-13

**Authors:** Gonzalo Sánchez-Barroso, Jaime González-Domínguez, Justo García-Sanz-Calcedo

**Affiliations:** Engineering Projects Area, Industrial Engineering School, University of Extremadura, 06007 Badajoz, Spain; gsm@unex.es (G.S.-B.); jaimegd@unex.es (J.G.-D.)

**Keywords:** green buildings, energy and environmental costs, domestic hot water (DHW), healthcare engineering, solar thermal energy

## Abstract

Hospitals need to prepare large amounts of domestic hot water (DHW) to develop their healthcare activity. The aim of this work was to analyse potential savings that can be achieved by installing solar thermal energy for production of domestic hot water in the hospitals of Extremadura (Spain). For this purpose, 25 hospitals between 533 and 87,118 m^2^ and between 15 and 529 beds were studied, three solar factor scenarios were simulated (0.70, 0.75 and 0.80) and the necessary investment and corresponding economic and environmental savings were calculated. Better economic results and energy ratios for 70% of solar contribution were obtained. These results show an average payback of 4.74 years (SD = 0.26) reaching 4.29 kWh/€ per year (SD = 0.20). Undertaking an investment of 674,423 €, 2,895,416 kWh/year of thermal energy could be generated with which to save both 145,933 € and 638 tons of CO_2_ per year. It was statistically demonstrated the priority of carrying out an installation with a solar factor of 70%, investing preferably in hospitals in Cáceres over those in Badajoz, especially in the public sector with more than 300 beds. These findings will provide hospital managers with useful information to make decisions on future investments.

## 1. Introduction

Hospitals are very energy-intensive buildings, because they operate continuously with high technical demands on supply and system reliability [1]. Water distribution systems in hospitals are an imperative service, especially for the preparation and distribution of domestic hot water (DHW) [2]. The Spanish hospital stock represents 3% of non-residential buildings [3] and is on average over 25 years old [4]. The equipment’s antiquity, combined with stringent demand specifications, means that hospitals are neither energy nor environmentally efficient [5]. Kolokotsa et al. [6] compiled technologies and strategies to promote energy savings and a reduction of CO_2_ emissions in hospital facilities and concluded that a series of measures can save up to 10% of primary energy while taking into account that the level of services provided in a hospital cannot be reduced [7].

Domestic hot water is an essential facility in hospitals which accounts for a large part of the thermal energy demand and represents approximately 15% of a hospital’s thermal consumption [8]. Moreover, hot water in a hospital is used mainly for sanitary purposes, laundry, kitchen and heated swimming pools for rehabilitation. It has been estimated that up to 50 kWh are needed to prepare 1 m^3^ of domestic hot water [9].

Adapting to new assistance services needs requires reforming and redesigning spaces which can influence the energy consumption of hospitals [10]. Thermal consumption in Spanish hospitals in 2017 was 5024.86 MWh, representing 11.1% of total consumption in the service sector [11]. The primary energy consumed in hospitals operating under normal operating conditions has been calculated as 0.27 MWh/m^2^, 9.99 MWh/worker and 34.61 MWh/bed [12]. Another study has gone deeper, creating indicators that relate thermal energy consumption to healthcare activity: 0.50 MWh/hospital discharge, 0.20 MWh/hospital stay, 1.60 MWh/surgery and 0.07 MWh/emergency action [13].

Southwest Spain receives an enormous amount of solar radiation that can be harnessed by installing renewable technologies to produce thermal energy for DHW. It has been proven that hospitals are a suitable type of building for these renewable energy installations, because they have a constant demand throughout the year [14]. Furthermore, these types of technical solutions contribute to improving the environmental efficiency of hospitals by reducing the CO_2_ emissions of their facilities. Studies have even shown that it is possible to achieve zero emissions by satisfying the energy needs of a hospital through hybridization of renewable energy sources [15].

The energy consumption of a DHW facility is related to the care activity carried out at the centre, the working hours, whether it has hospitalization services, its geographical location, etc. [16,17].

Several studies justify the suitability of applying solar thermal energy to meet the demand for DHW in buildings which have a constant demand throughout the year [18] such as hospitals. A series of annual, monthly, daily and even hourly indicators have been designed to control the operation of the system [19]. For example, it has been estimated that 8.5% of energy savings can be achieved by applying this technology to a hospital’s laundry service through simulation [20]. Other simulations have estimated the possible solar contribution using this technology at 61% for the Czech Republic [21].

Payback is often used as a financial performance index. Solar thermal showed three years lower payback (14 years versus 17 years) compared to solar photovoltaic for this application [21]. Hybridizing solar thermal technology with photovoltaic and biomass for DHW production is emerging; however, solar thermal is still more cost-effective today [22,23]. Research on architectural integration of renewable energy installations is trying to overcome the lack of roof area [24]. The technical-economic and environmental viability of installing solar collectors on facades is being evaluated [25,26]. Some studies have estimated the payback time of a single-family building investment at 15–20 years for different locations in Italy [27] and at 13.2 years on average for different Mediterranean cities [28].

Therefore, no author has so far studied in depth the economic and environmental savings possible in the DHW facilities of a representative group of hospitals. The aim of this paper was to analyse and quantify the potential for feasible savings through the installation of solar thermal energy for DHW production in the hospitals of Extremadura (Spain).

This work focused on calculating the energy savings for a heterogeneous set of hospitals, evaluating the corresponding economic and CO_2_ emissions savings to the atmosphere. This will complement the data that currently exist in the literature to apply tools for benchmarking the energy performance of buildings in order to direct efforts towards the most appropriate investments [29].

Given that all publicly owned buildings in Europe should be nearly zero energy buildings (NZEBs) from 2020 onwards, analysing the potential for savings in DHW facilities through the use of solar thermal energy in hospitals will provide useful information for making decisions on future investments [30].

## 2. Methodology

Twenty-five existing hospitals in the region of Extremadura, located in the southwest of Spain, were analysed. Their size ranged from 533 to 87,118 m^2^ and their beds from 15 to 529. After gathering functional parameters from each hospital, as explained below, those that did not have an available rooftop area to undertake an installation were discarded.

The following data were obtained from the Ministry of Health [31] to characterise each hospital: number of beds (NBs) and built surface area (BS). Roof surface (RS) was calculated by evaluating the construction plans of each building. The unusable surface (US) was then calculated by means of visual inspection of the RS. The ground on which it was not possible to install the solar collectors because of installed equipment and/or shading was taken into account. The value of US was deducted from RS thus obtaining the available surface (AS).

The hospitals were classified according to the following categories: geographical location (Badajoz or Cáceres), type of management (public or private) and size of the hospital (<120 beds, 120–300 beds and >300 beds). The study by García-Sanz-Calcedo et al. [32] on water consumption in the public hospitals of Extremadura justify the categorization of hospital size according to the NBs in the case of water consumption.

All hospitals are located between the latitude N 38.4° (H10) and N 40.0° (H22). Table 1 shows the functional characteristics of the analysed hospitals.

The DHW demand to be satisfied on a daily basis was calculated according to Spanish regulations [33] using Equation (1) adapted for hospitals and clinics:

D = 55 × NB(1)

where D is the reference demand for DHW at 60 °C (litres/day) and NB (units) the number of beds installed as the hospital’s fixed capacity.

The cold-water temperature was estimated for the different hospital locations according to UNE 94002:2005 [34] to calculate the energy required to raise it to the 60 °C reference. According to Spanish legislation [33], at least 70% of this energy must be provided by the Sun. Furthermore, two conditions were imposed against overheating that establish the upper limit of solar utilization: (1) not to produce 110% of the demand in any month and (2) not to exceed 100% of production for more than three months in a row.

A commercial model of solar collector was used, and the technical characteristics are indicated in Table 2. In this way, it was possible to obtain the number of solar thermal collectors (NSTCs) required.

Subsequently, the thermal energy generated by the solar field for DHW production during a calendar year (E), expressed in kWh per year, was calculated using MetaSol methodology [35]. MetaSol methodology uses solar radiation data collected hourly in the “Atlas of Solar Radiation in Spain” by the State Agency of Meteorology during the period 1983–2005 [36]. Its calculation procedure is based on curves obtained by the f-chart statistical method [37] from results of more than 69,000 dynamic simulations performed in TRNSYS [38]. Over 800,000 data are obtained on a monthly basis which constitute the information used to generate correlations.

An iterative process on the MetaSol methodology was carried out to check the upper limit of possible solar contribution, taking into account the lower and upper limitations described. A solar factor greater than 0.8 could not be achieved; therefore, three situations were considered to be feasible and representative of the percentage of solar contribution (solar fraction, f_s_) that can be achieved: 70% (minimum), 75% (intermediate) and 80% (maximum). For each level of this factor, the necessary capturing area was estimated, knowing the value of global horizontal solar radiation for each location and the conversion factor *k* that relates it to the radiation on the tilted plane for each latitude (embedded in MetaSol methodology).

Next, the amount of CO_2_ not emitted was estimated with Equation (2), considering that the E should have been produced by the DHW facility currently installed in the hospitals.

CO_2_ eq = E × CF (2)

where CF is a conversion factor for the technology currently installed in each hospital to the amount of CO_2_ equivalent indicated by the Spanish Ministry of Development [39].

Then, the capital expenditure (CAPEX) for the physical construction of the planned facilities was calculated according to Equation (3) after contact with suppliers, and operational expenditure (OPEX) was calculated annually as 2% of the CAPEX.

CAPEX = NSTC × 580 €/u + NSTC/32 × 3000 €/u(3)

where CAPEX is the value of the material investment in euros (€) and NSTC is the number of solar thermal collectors installed (units).

Finally, the annual savings attributed to DHW production facilities (S), were calculated, estimating a value of 0.055 €/kWh per year. Ten years of the plant life were proposed. With these data it was possible to obtain the economic payback index for each case. Possible government aid for the investment was not taken into account which would further reduce the payback.

**Figure 1:**
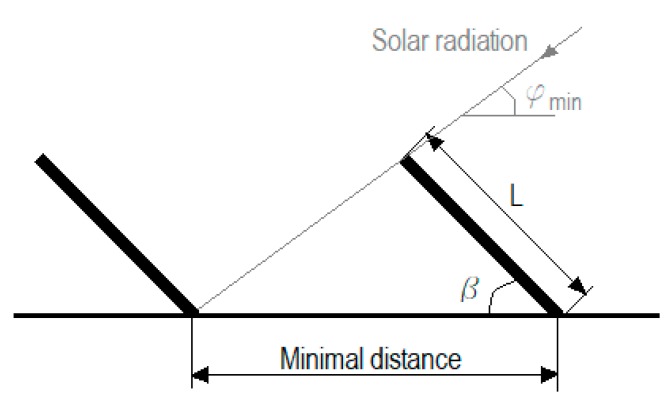
Diagram to calculate the minimum distance between collectors on a flat surface.

Additionally, the area occupied by each thermal collector unit was calculated using Equation (4) with the measurements of the commercial model selected with an inclination between 48.4° and 50° above the horizontal (latitude increased by 10°) and zero azimuth, taking into account the minimization of shadow production between them, as shown in Figure 1.

Minimal distance =·L × cos β + (L × sin β)/tg φ_min_ (4)

where L (mm) is the length of the solar collector, β (°) is the tilt angle that the solar collector forms with the horizontal surface and φ_min_ (°) is the minimum solar declination (angle between sun–earth centreline and the equatorial plane).

Pearson’s coefficient was used to check whether there was a relationship among energy results and functional parameters of a hospital. This was a measure of the linear correlation among quantitative variables used to determine if this correlation was significant with a 95% significance level. Significant correlation between energy generated and the built surface area of hospitals was the only one noticed. A linear regression model was proposed to determine a mathematical equation that relates them. This model was validated after verifying that its residuals complied with the premises of independence, normality and homoscedasticity.

Independence implies that there was no correlation among the residues of the intervening variables. For this purpose, the Durbin–Watson test was used which takes values between 0 and 4, with those around 2 being acceptable for ensuring the lack of correlation among residues [40]. The residues were checked for normality with a histogram of the standardized residues. The homoscedasticity of the model variables was verified by graphical methods using the residual versus fitted plot. Values between −2 and +2 were taken as valid and no association pattern was detected between them.

Samples were tested for normality with Shapiro–Wilk (*N* < 30) and for homogeneity of variances with the Levene test. The non-parametric Kruskal–Wallis test was applied to determine if there was a significant difference in average values among groups.

## 3. Results

### 3.1. Energetic, Environmental and Economic Results

The NSTCs required to achieve the expected solar factor in each hospital is shown in Table 3. Due to the unavailability of rooftop area, there are hospitals that do not have an assigned value (number 11, 12, 15, 16 and 23). For each level of solar factor, NSTCs ranges from 6 to 138 for fs = 0.70, for fs = 0.75 the minimum is 7 and the maximum is 160, and for fs = 0.80 it ranges from 9 to 204.

**Figure 2:**
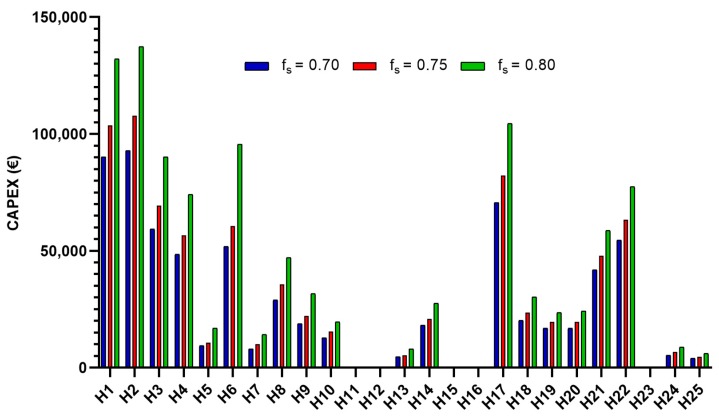
Capital expenditure according to the solar factor for each hospital.

The value of the investment to be assumed by each hospital according to the level of solar contribution desired to carry out these projects is shown in Figure 2. The CAPEX ranges from 4042.50 € for a hospital with 24 beds and an fs = 0.70, to 137,445.00 € for a hospital with 529 beds and an fs = 0.80. For each level of solar factor, the full investment would be: 674,423 € (OPEX = 13,488.48 €/year), 786,266 € (OPEX = 15,725.33 €/year) and 1,028,142 € (OPEX = 20,562.85 €/year), respectively.

**Figure 3:**
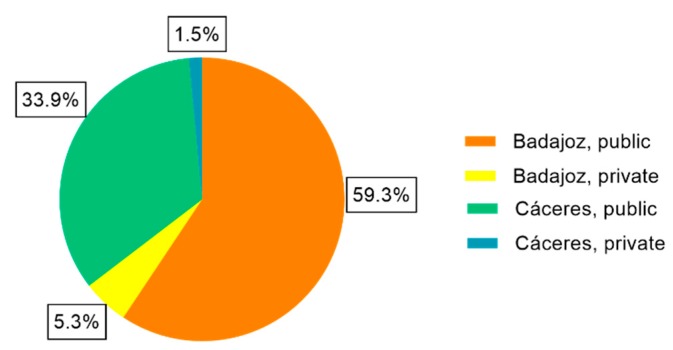
Proportion of thermal energy generated based on province and type of management.

Adding the individual results of each hospital, 2,895,417 kWh/year will be generated for the minimum solar contribution (70%), 3,068,977 kWh/year for the 75% contribution and 3,239,578 kWh/year for the 80% solar factor which means an annual economic savings of 145,933.41 €, 153,503.46 € and 157,723.94 €, respectively. The proportion of thermal energy according to province and type of management that will be generated by the DHW installations of the analysed hospitals is shown in Figure 3.

**Figure 4:**
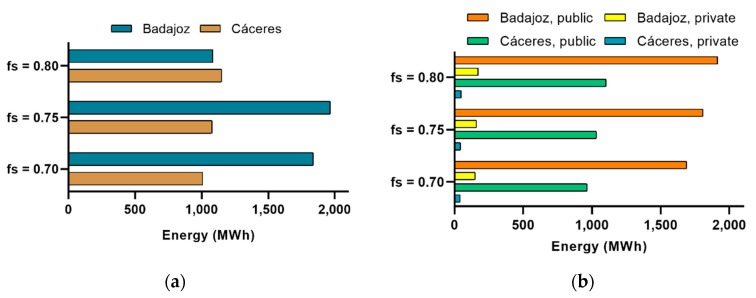
Energy generated by hospitals according to (**a**) province and (**b**) both province and type of management.

Separating by provinces and by type of management, the results shown in Figure 4 were obtained. For each level of solar contribution, the hospitals in Badajoz would generate 82% more on average than those in Cáceres. The difference between public and private was much greater, being on average 1382.5% in favour of public ones.

**Figure 5:**
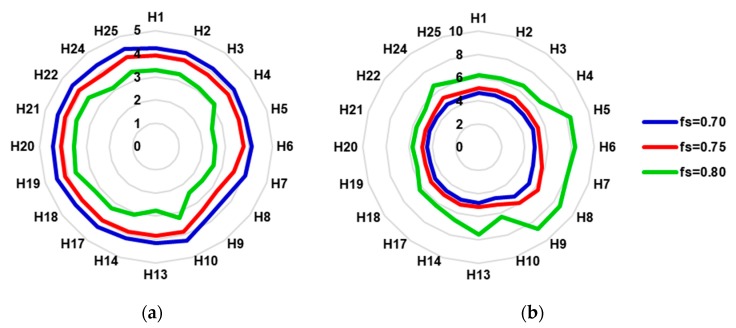
(**a**) Ratio: energy/CAPEX (kWh/€ per year); (**b**) payback calculated for each investment (years).

In all cases, the ratio of energy generated to CAPEX decreases as the solar factor increases. For the minimum solar contribution, we have an average of 4.29 kWh/€ per year (standard deviation, SD = 0.20) for fs = 0.70, for the 75% contribution we calculated 3.90 kWh/€ per year (SD = 0.23) and for the highest solar factor, 3.15 kWh/€ per year (SD = 0.39). The value of this ratio for each hospital can be seen in Figure 5a.

The payback for each investment is shown in Figure 5b. The increase in the average global payback with respect to fs = 0.70 is 10.55% and 43.46% for fs = 0.75 and for fs = 0.80, respectively; in some cases reaching 72.9% (hospital number 6) and 68.7% (hospital number 5). If all investments were undertaken, it would mean a payback of 4.74 years (SD = 0.26 years) for a solar contribution of 70%, 5.24 years (SD = 0.38 years) for 75% and 6.80 years (SD = 1.07 years) for 80%.

Separating by province, providing 70% of the demand continues to be the most appropriate as the lowest payback value is presented by the hospitals of Cáceres (average of 4.52 years) followed by those of Badajoz (average of 4.89 years). In the next level of solar contribution, those of Caceres present a payback of 0.46 years less than those of Badajoz. For the highest solar factor value, this difference increases to 1.40 years. The worst payback results were obtained for the hospitals of Badajoz if an installation is projected for fs = 0.80.

According to the hospital size NB-based classification, the best results were obtained for type C (NB > 300) with a payback of 4.66 years (SD = 0.12 years) for fs = 0.70. The worst results were for type B (NB = 120–300) with 7.32 years (SD = 1.44 years) and solar factor 0.80. For the intermediate step of solar contribution, the best results were again for type C with 5.09 years (SD = 0.15 years) and the worst results were for type B with 5.43 years (SD = 0.64 years).

Assuming that the installed solar thermal system replaces the current technology of each hospital, it is estimated that annual CO_2_ emissions range from 637,991 kg for the lowest solar factor to 721,016 kg for the highest one and 678,462 kg for the intermediate level. The average savings of hospitals according to their size per year is shown in Table 4.

### 3.2. Relationship between Built Surface Area and Produced Energy

**Figure 6:**
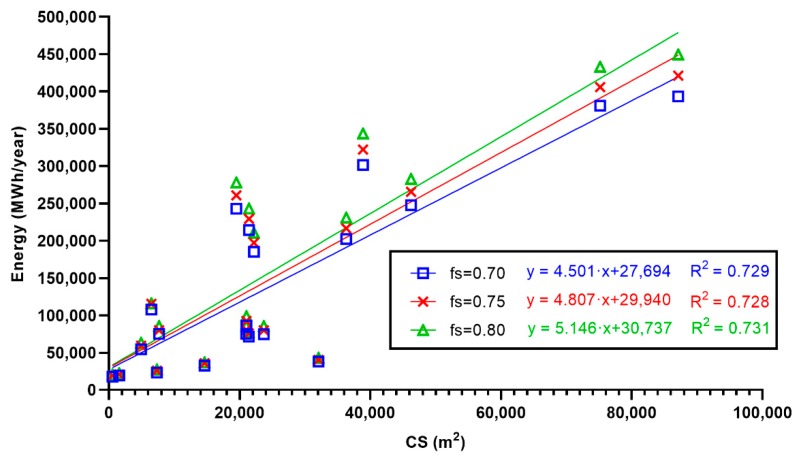
Energy contributed annually by built surface area.

The energy that can be produced was estimated as a function of the built surface area according to the desired solar fraction. The results for Pearson’s (R) correlation test are shown in Table 5, and the significance level indicates that they were indeed correlated. It was then proposed to establish the regression model shown in Figure 6.

Values of 2.641, 2.642 and 2.624 were obtained from the Durbin–Watson test for fs = 0.70, fs = 0.75 and fs = 0.80, respectively. Values close to 2 were obtained thus confirming the independence among residuals.

**Figure 7:**
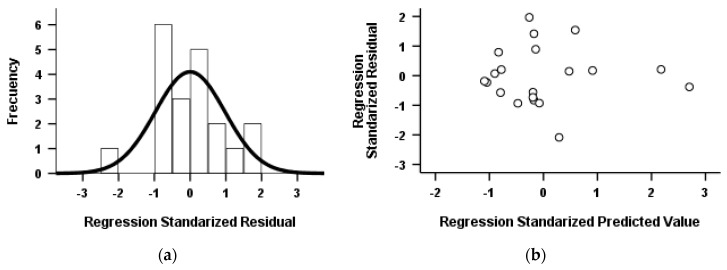
Graphs to verifying both normality and independence of residues: (**a**) histogram of the standardized residual and (**b**) scatterplot of the standardized residuals with the standardized predicted values.

Figure 7 shows (a) the test for normality of the residues and (b) the relationship among standardized forecasts (X) and standardized residues (Y). It can be seen that (a) most of the points are in the interval [–2,2], and (b) there is no pattern of association among variables which means that there is homogeneity of variances.

Therefore, the proposed linear regression model was adequate to predict the value of savings (or energy contributed by the facility) as a function of the built surface area of a hospital. The key performance indicator (KPI) shown in Equation (5) was constructed to help adjust the plant equipment at start-up:

KPI #1 = Supplied Energy/CS.(5)

### 3.3. Prioritisation of Investment Alternatives

The significance level of Shapiro–Wilk’s test for payback was 0.032 for fs = 0.70, 0.003 for fs = 0.75 and 0.007 for fs = 0.80. If any case had a *p*-value > 0.05, consequently, it was not verified that the samples followed a normal distribution for the three levels of solar fraction. After applying the Kruskal–Wallis test, a *p*-value of 1.5 × 10^−10^ was obtained (K-W’s H was 45.201 and df = 2), so there was a significant difference in the mean payback among the three levels of solar fraction.

When the data were categorized according to province and solar factor, the significance values in Table 6 were obtained after applying S–W’s test. Two of the three categories for Cáceres were the only ones that followed a normal distribution, so the K–W’s test was performed. The test results show *p*-value of 4.9 × 10^−10^ (K–W’s H was 52.219 and df = 5), so there was a significant difference among groups.

When we performed the same procedure for type of management, we obtained the significance levels in Table 7. There were samples that did not fit a normal distribution, so a K–W’s test was done. A *p*-value of 1.2 × 10^−8^ was obtained (K–W’s H was 45.468 and df = 5). This implies that there was a significant difference among the average of the six groups.

According to the hospital size classification by number of beds, the significance value in Table 8 was obtained after applying S–W’s test. There were two cases of significance value lower than 0.05, so these did not follow a normal distribution. Therefore, K–W’s test was performed. The test results showed a *p*-value of 2.9 × 10^−7^ (K–W’s H was 45.502 and df = 8), so there was a significant difference among groups.

**Figure 8:**
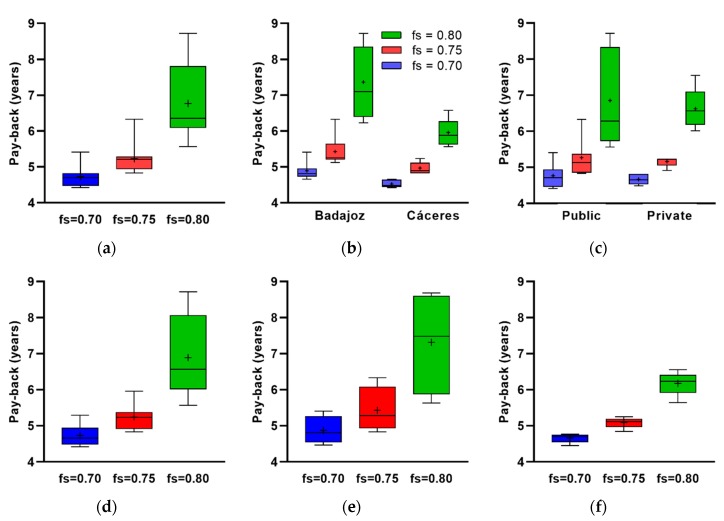
Payback: (**a**) global, (**b**) by province, (**c**) by type of management, (**d**) NB < 120, (**e**) 120 ≤ NB ≤ 300 and (**f**) NB > 300, according to solar fraction.

These analyses are shown graphically in Figure 8, where the boxes of the different groups do not overlap with all the others which means that there is a difference among them. In all cases, better results can be detected for fs = 0.70. In general, it can be said that it is more attractive to invest to satisfy 70% of the demand, more profitable in the hospitals of Cáceres than those of Badajoz and the priority cannot be determined graphically according to the type of management. Depending on the size of the hospital, it also remains more interesting to invest to achieve 70% of demand. The differences in the payback rate among sizes within this solar factor were not remarkable. However, the most profitable were those in NB > 300 category.

**Figure 9:**
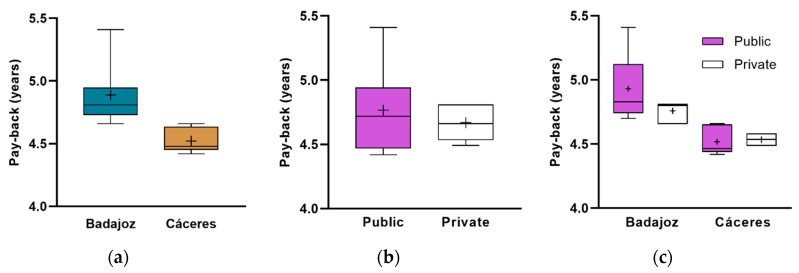

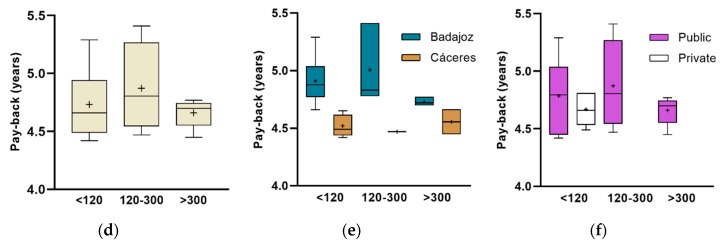
Payback for solar contribution of 70% classified by (**a**) province, (**b**) management, (**c**) both of them, (**d**) NB, (**e**) NB and province and (**f**) NB and type of management (there are not private hospitals in NB > 120 categories).

The analysis was specified for 70% of solar contribution, since it was the best investment considering its payback. Figure 9 shows this index by province, by type of management, by both and by NB-based classification. It is more interesting in the hospitals of Cáceres than those of Badajoz. According to the type of management is indifferent. Combining both, investments should be prioritized over public hospitals in Cáceres despite having a similar average payback, because they are more numerous than private ones. As per NB-based classification, the most profitable investments would be in NB > 300 hospitals; it would be quite similar in Cáceres for NB < 120 and NB > 300; finally, priority by size and type of management could not be determined graphically.

## 4. Discussion

Throughout the research, it was proven that hospitals are among the most cost-effective tertiary sector buildings for implementing solar thermal hot water production systems. The use of the facilities 24 h/365 days makes the return on investment pay off in a short period of time [41]. In addition, the use of solar energy to produce DHW avoids constant variations in the price of gas and electricity, which tends to be upward [42], which is important for adjusting the hospital’s annual budgets.

Positioning panels at an inclination 10° higher than the geographical latitude allows for increased capture during months of lower solar radiation [43]. The solar fraction will decrease in summer, consequently. Nevertheless, less energy is required to bridge the thermal gap in summer than winter, and the support system’s cost will be reduced. Consequently, this arrangement will be more cost-effective and auxiliary energy consumption will be reduced in favour of the solar contribution. Another advantage of implementing solar thermal energy for DHW generation is that the internal space available in the hospital will be increased, because the infrastructure is located on the hospital’s rooftops, usually without defined use [44].

It was noted that the NSTC does not increase proportionally with increasing solar fraction. For all cases studied, the demand for DHW was completely satisfied with the solar capture during the months of June, July and August due to the enormous amount of solar radiation received. During the rest of the year, it would be necessary to install a greater number of solar collectors to increase the overall solar contribution due to the significantly lower radiation.

It was also found that for the solar factor of 0.70, the best payback values were obtained both for the overall investment and by separating the hospitals by province and by management. The hospitals in the province of Cáceres presented better payback data at all levels.

Investment policy in hospitals tends to prioritize more urgent actions, leaving aside investments in energy efficiency [45]. However, there are alternative sources of financing such as energy service companies (ESCO) or public–private partnership (PPP) contracts [46]. Crowdfunding has also been tried [47].

The modernization of hospital buildings, both public and private, to promote energy efficiency is essential [48]. The incorporation of government subsidies and incentives can make investment even cheaper, encouraging rapid amortization of facilities [49].

District generation can be a suitable solution, because it allows a better use of energy. It has been found that most of the EU28 member states have good conditions for district heating [50]. Hybrid photovoltaic-thermal systems are also interesting, because they have the ability to convert solar energy into electricity and into thermal energy simultaneously [51] and can be used to meet, to a large extent, the energy demand of hospitals [52]. Another interesting option is to use solar energy to produce cold by means of absorption equipment [53].

One option to further reduce CO_2_ emissions to the atmosphere is to supplement production with biomass which will improve the environmental outlook, although facilities are made more sophisticated by increasing maintenance costs [54].

It is essential monitoring and modifying operation set points of different pumped systems according to the results obtained by calculation [55]. Another important consideration can be the use of available energy resources in the hospital centre, produced by other systems or equipment which must be registered in energy audit reports [56,57].

In any case, the elimination of *Legionella* has to be guaranteed [58], since the hospital is a very sensitive building to *Legionella* infection due to the fragility of the patients admitted [59,60].

This paper is useful for hospital managers to assess investment, savings and environmental benefits. The geographical location of the hospitals studied and the applicable regulatory requirements can be established as a limitation; nevertheless, the methodology used can be extended to other regions.

Future work should focus on analysing the feasibility of introducing other renewable energy sources to support the solar thermal energy production facility.

## 5. Conclusions

This paper reported all the energy, economic and environmental calculations related to the implementation of solar thermal energy to prepare DHW in the 25 hospitals of Extremadura (Spain). An exhaustive analysis of the decomposed calculations for different classifications (i.e., province, management and size) was carried out for three levels of solar contribution (i.e., 70%, 75% and 80%).

Better overall results were obtained for investing in an installation that covers 70% of DHW demand with solar thermal energy. For all cases studied, this level of solar contribution shows the lowest payback values (mean of 4.74 years and SD = 0.26 years). Furthermore, the highest ratio of thermal energy for each monetary unit invested (mean 4.29 kWh/€ per year and SD = 0.20 kWh/€ per year) will be generated. Carrying out an investment of 674,423 € (plus an OPEX of 13,488.48 €/year), 2,895,416 kWh/year of thermal energy could be generated with which to save both 145,933.41 and 637.99 tons of CO_2_ per year.

For this level of solar factor: on the one hand, Badajoz hospitals require an investment of 444,001.25 € with OPEX of 8,880.03 €/year to produce 1,870,439 kWh/year with a ratio of 4.21 kWh/€ (SD = 0.17 kWh/€). Annual savings of 94,127.05 € and 411.51 tons CO_2_ are achieved which means an average payback of 4.89 years (SD = 0.23 years). On the other hand, the hospitals of Cáceres would produce 1,024,978 kWh/year of thermal energy with an index of 4.45 kWh/€ (SD = 0.08 kWh/€) if an investment of 230,422.50 € with an OPEX of 4608.45 €/year is made. The annual savings are 51,806.36 € and 226.48 tons CO_2_ which translates into an average payback of 4.52 years (SD = 0.09 years).

It has been statistically proven that the investment is more profitable in the hospitals of Cáceres than those in Badajoz. The priority cannot be determined according to type of management; however, managers should give priority to investment in the public hospitals of Cáceres considering both province, type of management and size.

## Figures and Tables

**Table 1:** Functional characteristics of the hospitals under study.

Hospital	Province	Type of Management	NB	BS ^1^ (m^2^)	RS ^2^ (m^2^)	US ^3^ (m^2^)	AS ^4^ (m^2^)
H1	Badajoz	Public	510	75,173	16,314	3900	12,414
H2	Badajoz	Public	529	87,118	20,731	6374	14,357
H3	Badajoz	Public	331	46,207	8,145	4600	3545
H4	Badajoz	Public	270	36,308	18,446	6080	12,366
H5	Badajoz	Public	50	32,074	9992	1200	8722
H6	Badajoz	Public	284	21,439	14,943	2500	12,443
H7	Badajoz	Public	43	14,630	12,336	3850	8386
H8	Badajoz	Public	136	6486	5667	2300	3367
H9	Badajoz	Public	91	21,381	9842	7492	2350
H10	Badajoz	Private	74	4903	1016	816	200
H11	Badajoz	Private	20	5025	797	797	0
H12	Badajoz	Private	101	3130	1412	1412	0
H13	Badajoz	Private	26	1549	795	105	690
H14	Badajoz	Private	100	7678	3189	430	2759
H15	Badajoz	Private	29	2950	2293	2293	0
H16	Badajoz	Private	15	2556	586	586	0
H17	Cáceres	Public	404	38,880	11,187	3240	7947
H18	Cáceres	Public	116	21,024	6297	4747	1550
H19	Cáceres	Public	103	20,998	6447	1732	4715
H20	Cáceres	Public	102	23,702	7416	967	6449
H21	Cáceres	Public	250	22,192	6761	1280	5481
H22	Cáceres	Public	330	19,489	6187	1300	3887
H23	Cáceres	Private	35	2920	646	646	0
H24	Cáceres	Private	32	7320	2077	1414	663
H25	Cáceres	Private	24	533	549	154	395

^1^ BS: built surface area; ^2^ RS: rooftop surface; ^3^ US: unusable surface; ^4^ AS: Available surface.

**Table 2:** Technical characteristics of the solar thermal collectors employed.

Dimensions	Absorber Surface	Absorptance	Zero-Loss Efficiency	1st Order Coefficient	2nd Order Coefficient
1753 × 1147 × 87 mm	3.76 m^2^	95%	0.724	3.860 W/m^2^K	0.017 W/m^2^K

**Table 3:** Number of solar thermal collectors for each hospital according to the solar factor.

**Solar Factor**	**H1**	**H2**	**H3**	**H4**	**H5**	**H6**	**H7**	**H8**	**H9**	**H10**
70%	134	138	88	72	14	77	12	43	28	19
75%	154	160	103	84	16	90	15	53	33	23
80%	196	204	134	110	25	142	21	70	47	29
**Solar factor**	**H13**	**H14**	**H17**	**H18**	**H19**	**H20**	**H21**	**H22**	**H24**	**H25**
70%	7	27	105	30	25	25	62	81	8	6
75%	8	31	122	35	29	29	71	94	10	7
80%	12	41	155	45	35	36	87	115	13	9

**Table 4:** Amount of CO_2_ (ton/year) not emitted to the atmosphere.

Category		Fs = 0.70	Fs = 0.75	Fs = 0.80
NB < 120	Mean	11,466.18	12,332.27	13,080.82
	SD	5772.66	6152.41	6537.60
120 ≤ NB ≤ 300	Mean	39,267.50	42,124.00	44,541.50
	SD	10,581.70	11,369.59	12,870.84
NB > 300	Mean	69,358.60	74,422.20	79,592.20
	SD	15,829.35	16,816.71	18,052.30

**Table 5:** Pearson’s correlation test between built surface area and energy input according to solar fraction.

		Energy Supplied		
		Fs = 0.70	Fs = 0.75	Fs = 0.80
CS	R	0.854	0.853	0.855
	Significance	2 × 10^−6^	2 × 10^−6^	2 × 10^−6^

**Table 6:** Shapiro–Wilk’s test results for data categorized by province.

Province Solar Factor	Badajoz			Cáceres		
	70%	75%	80%	70%	75%	80%
Significance	0.010	0.003	0.030	0.116 *	0.046	0.295 *

* Normal distribution.

**Table 7:** Shapiro–Wilk’s test results for data categorized by type of management.

Type of Management Solar Factor	Public			Private		
	70%	75%	80%	70%	75%	80%
Significance	0.103 *	0.023	0.009	0.418 *	0.001	0.481

* Normal distribution.

**Table 8:** Shapiro–Wilk’s test results for data categorized by NB-based size.

Size	NB < 120			120 ≤ NB ≤ 300			NB > 300		
Solar Factor	70%	75%	80%	70%	75%	80%	70%	75%	80%
Significance	0.428	0.174	0.243	0.266	0.135	0.175	0.971	0.034 *	0.020 *

* Non-normal distribution.
